# Supplementary figures and images for: Epistasis among clustered lineage-specific amino acid substitutions in the Drosophila Trio protein
Source: PLoS Genet. 2026 Jun 3;22(6):e1012175. doi: 10.1371/journal.pgen.1012175 (PMC13258152; doi:10.1371/journal.pgen.1012175)

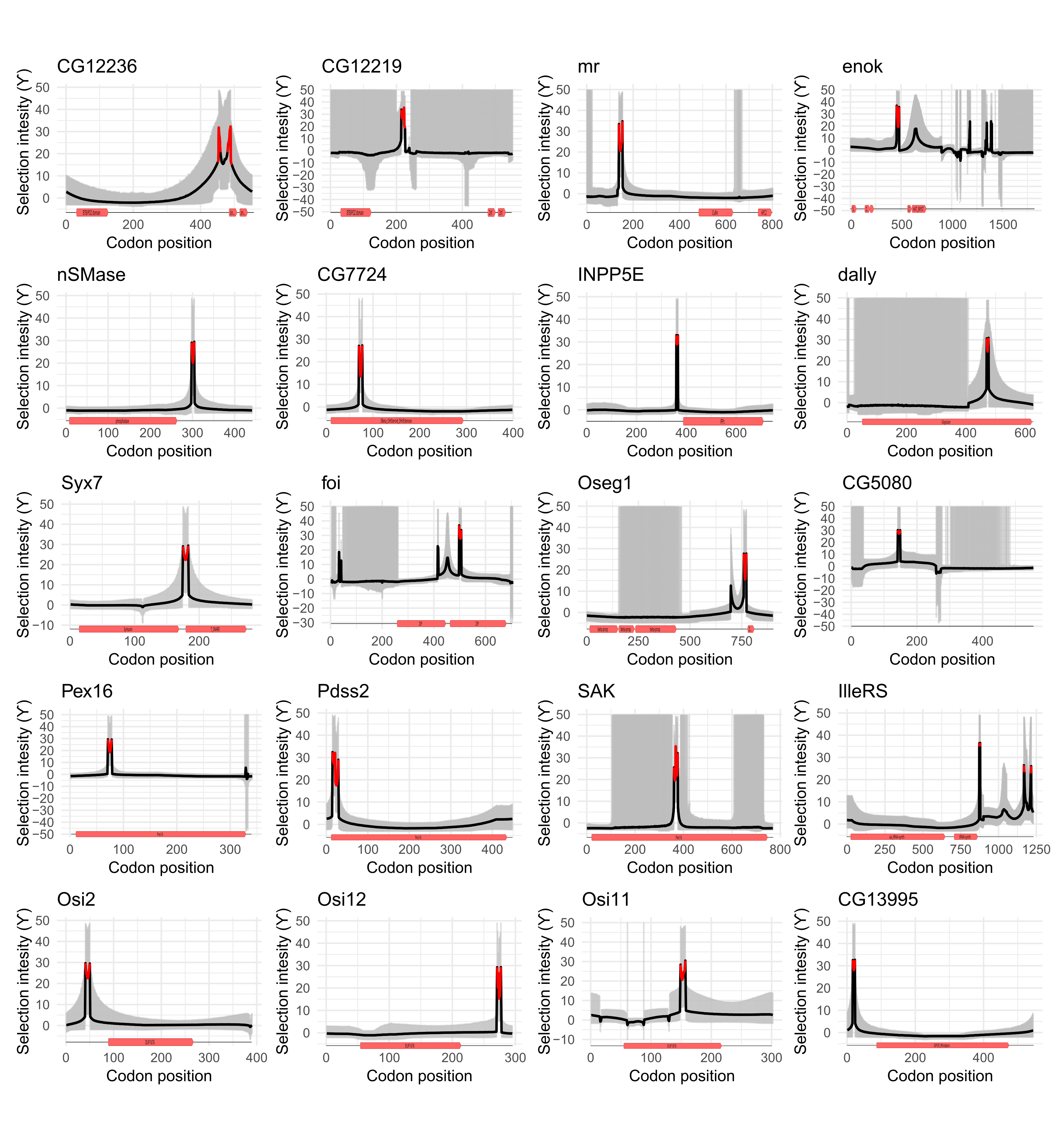

Supplement: S1 Fig — Plotted are profiles of selection intensity (γ = 2Ns) across 20 proteins inferred with MASS-PRF (1). The black line corresponds to the model-averaged γ and the grey areas indicate 95% model uncertainty interval. Red lines indicate regions for which the 95% lower bound of γ > 4, which corresponds to a false positive rate of < 0.1 (see Methods). Protein models are represented below each selection intensity profile. Functional domains are represented by red boxes on the x-axis. (TIFF) [file pgen.1012175.s004.tiff]

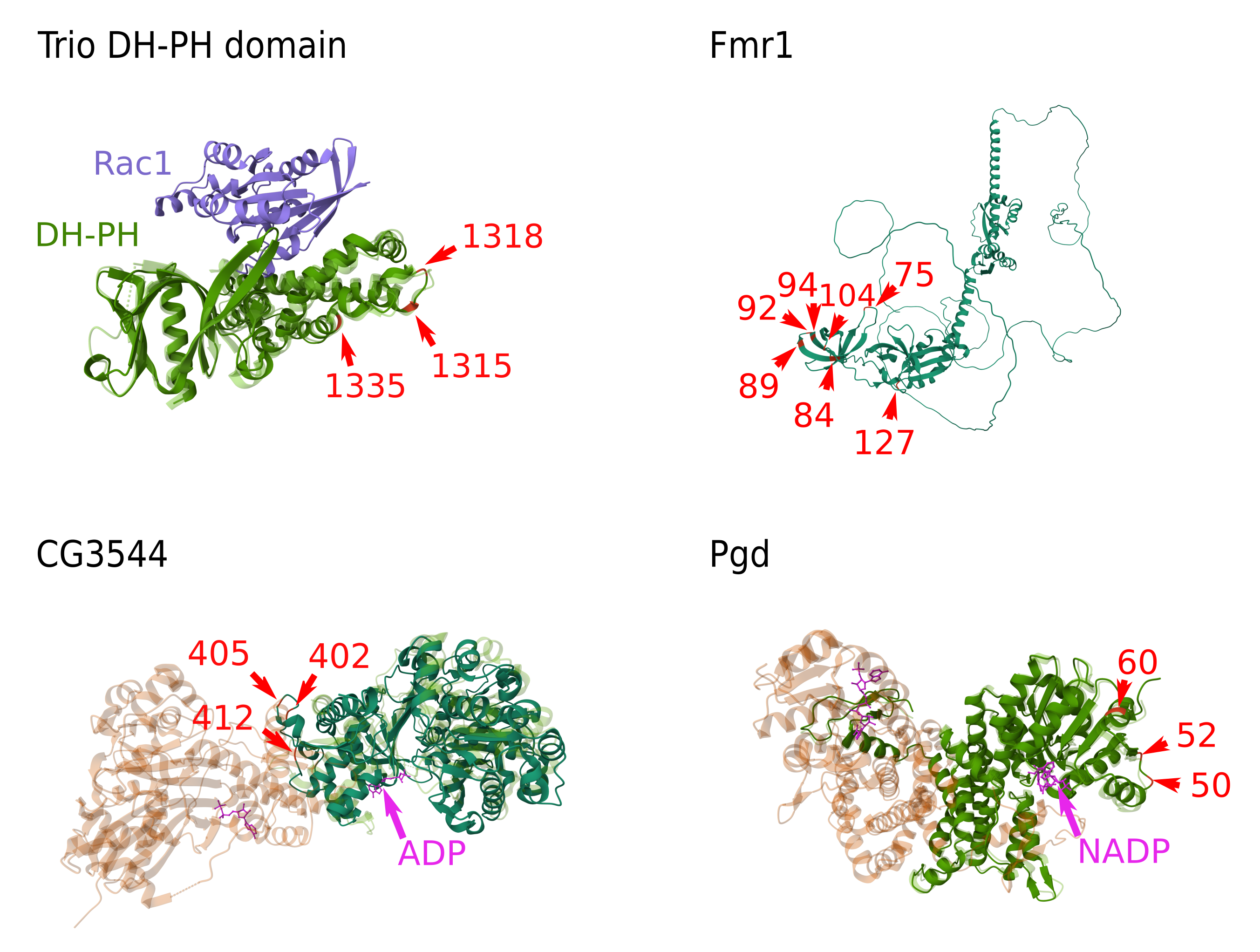

Supplement: S2 Fig — In this version, AlphaFold predictions of the 3D structure of the D. melanogaster proteins are superimposed onto homologous PDB structures (See Fig 1 for more details). (TIFF) [file pgen.1012175.s005.tiff]

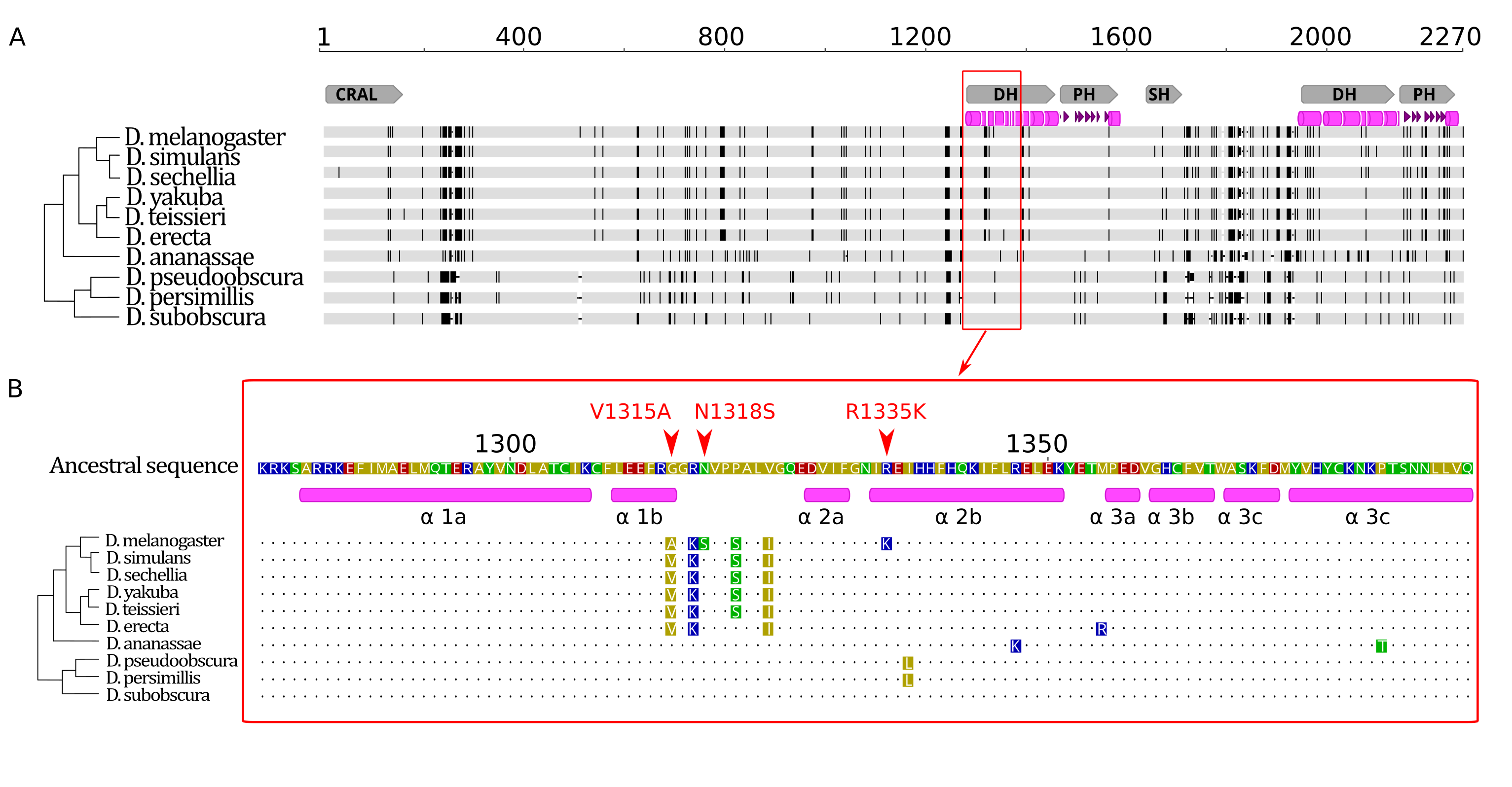

Supplement: S3 Fig — (A) Alignments of the full protein sequence with large insertion-deletion differences removed. Differences with the inferred ancestral sequence are represented in black and identity in grey. Functional domains are represented by grey boxes. (B) A zoom-in of the Trio D. melanogaster lineage-specific cluster region. Amino acids are colored according to their polarity (yellow: non-polar; green: polar; red: polar, acid; blue: polar, basic). Dots represent identity with the inferred ancestral sequence. Positions of alpha-helices are based on the human structure (PDB 7SJ4, (2)). Species tree topology is taken from references (3,4). Branch lengths are not at scale. The multiple alignment was generated using muscle (v 3.8.425), as implemented in Geneious Prime (v 2019.0.4). The D. melanogaster-D. subobscura ancestral Trio sequence was inferred with the codeml function of PAML (v 4.9; (5)) using protein sequences of D. melanogaster, D. simulans, D. yakuba, D. ananassae, D. pseudoobscura, D. willistoni, D. virilis and the corresponding species tree. (TIFF) [file pgen.1012175.s006.tiff]

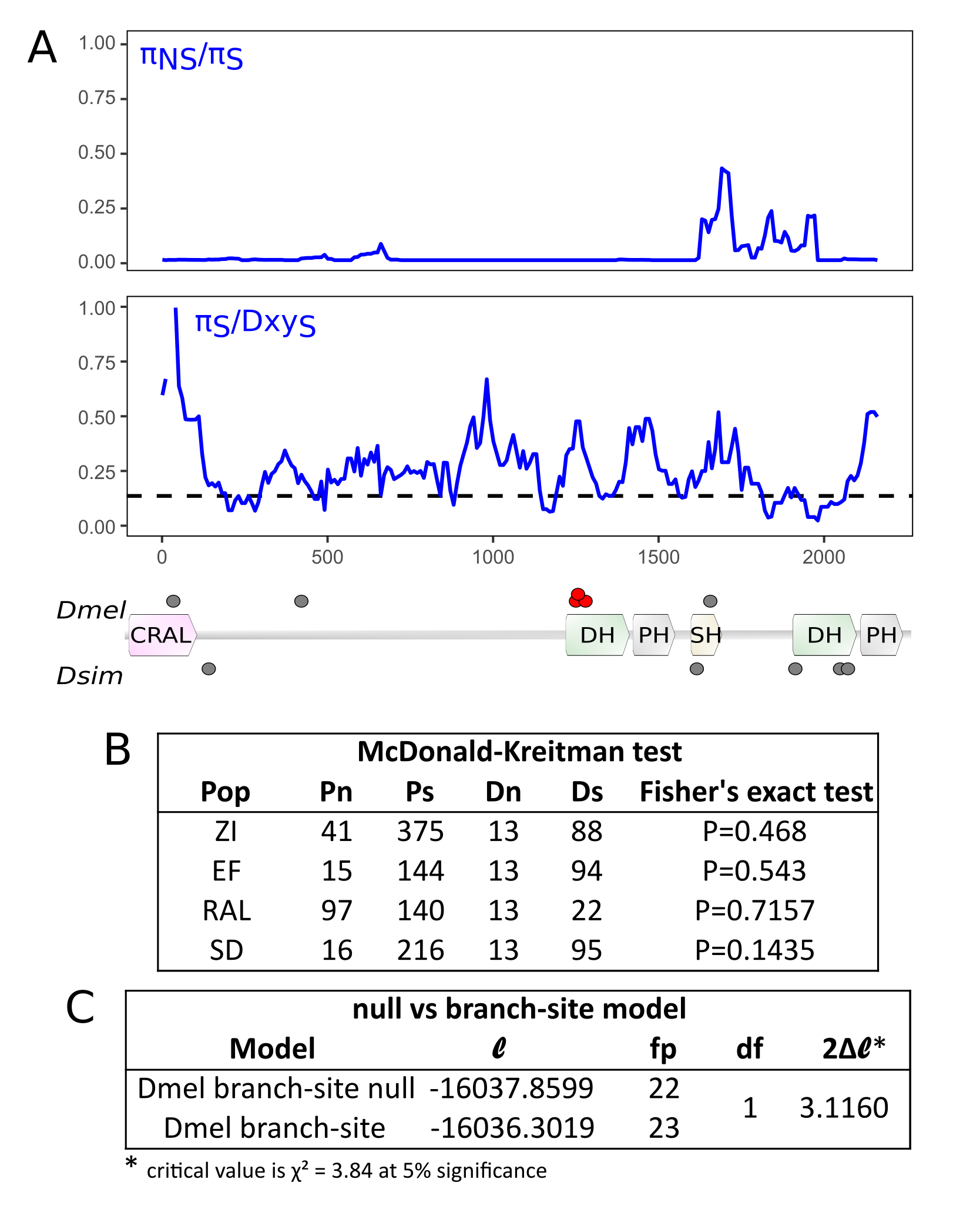

Supplement: S4 Fig — (A) Trio exhibits exceptionally strong sequence constraint and there is no indication of a selective sweep associated with the clustered D. melanogaster lineage-specific substitutions (red dots). Plotted are a measures of selective protein sequence constraint (πNS/πS) and linked synonymous site diversity (πS/Dxy,S). S: synonymous; NS: nonsynonymous; in both cases, only 4-fold degenerate codons were used. Diversity π and divergence Dxy were calculated in overlapping sliding windows of 100 codons with 10 codon steps using SNPGenie (6). π was calculated using individuals from the Raleigh population (n=210) (7) and divergence Dxy using the D. melanogaster (GCA_000001215.4) and the D. simulans (GCA_016746395.2) genome reference sequences. Black dashed lines correspond to the average value of p/Dxy for the middle of chromosome arm 3R in the Raleigh population (8). (B) McDonald-Kreitman tests performed using DNAsp6 (9) using several D. melanogaster populations are not significant. ZI: Zimbabwe; EF: Ethiopia; RAL: Raleigh; SD: South Africa (7). (C) Branch-site model performed with PAML (v 4.9; (5, 10)) using the Drosophila species tree does not show sign of selection in the D. melanogaster branch. l: log-likelihood; fp: free parameters; df: degree of freedom; LTR statistics 2Δl = 2(l1- l0). (TIFF) [file pgen.1012175.s007.tiff]

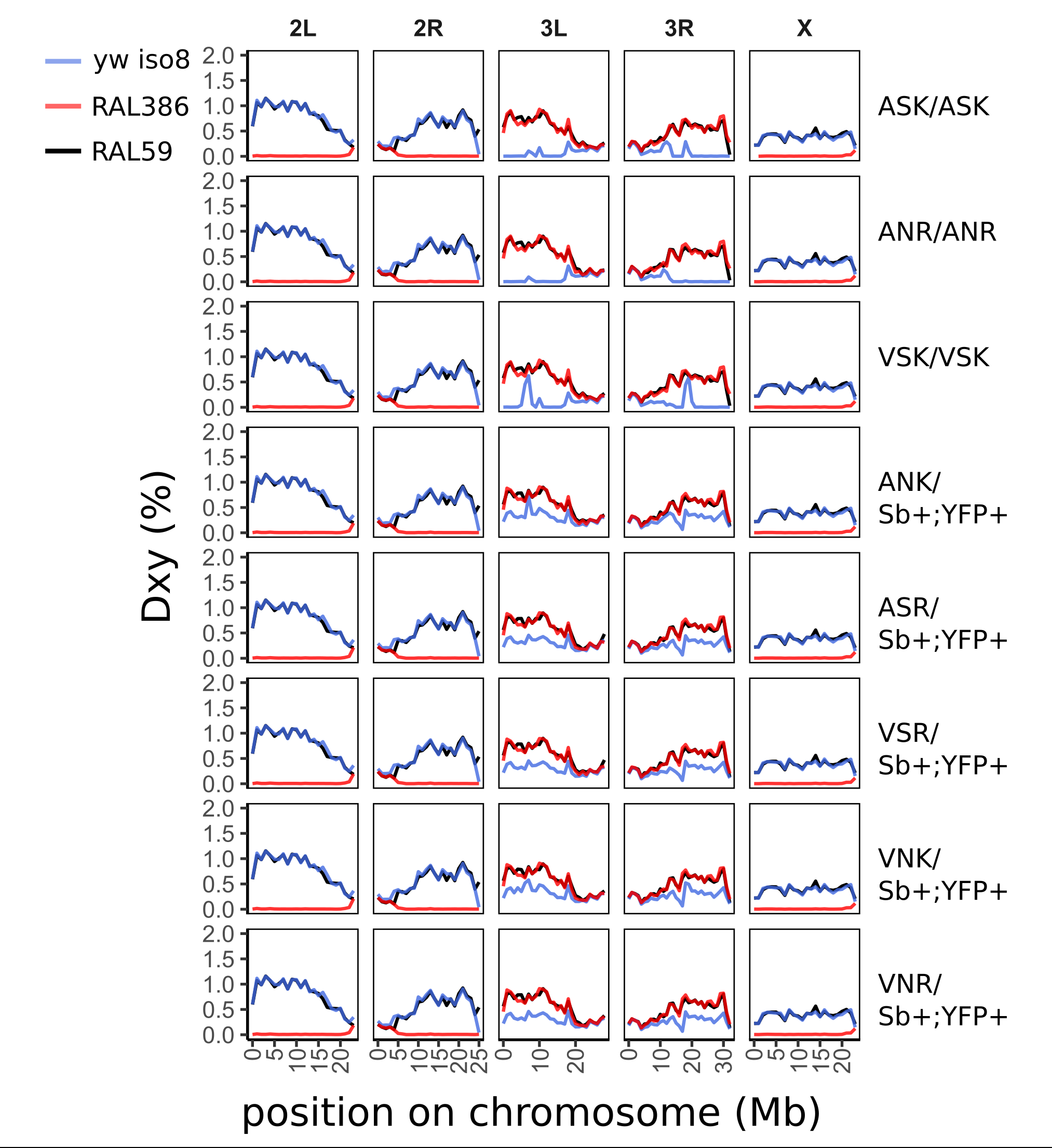

Supplement: S5 Fig — Average pairwise divergence (Dxy) between engineered strains and RAL386 (red line), yw iso8 (blue line) and RAL59 (black line) was calculated in 1 Mbp non-overlapping windows along chromosome arms (see Methods). (TIFF) [file pgen.1012175.s008.tiff]

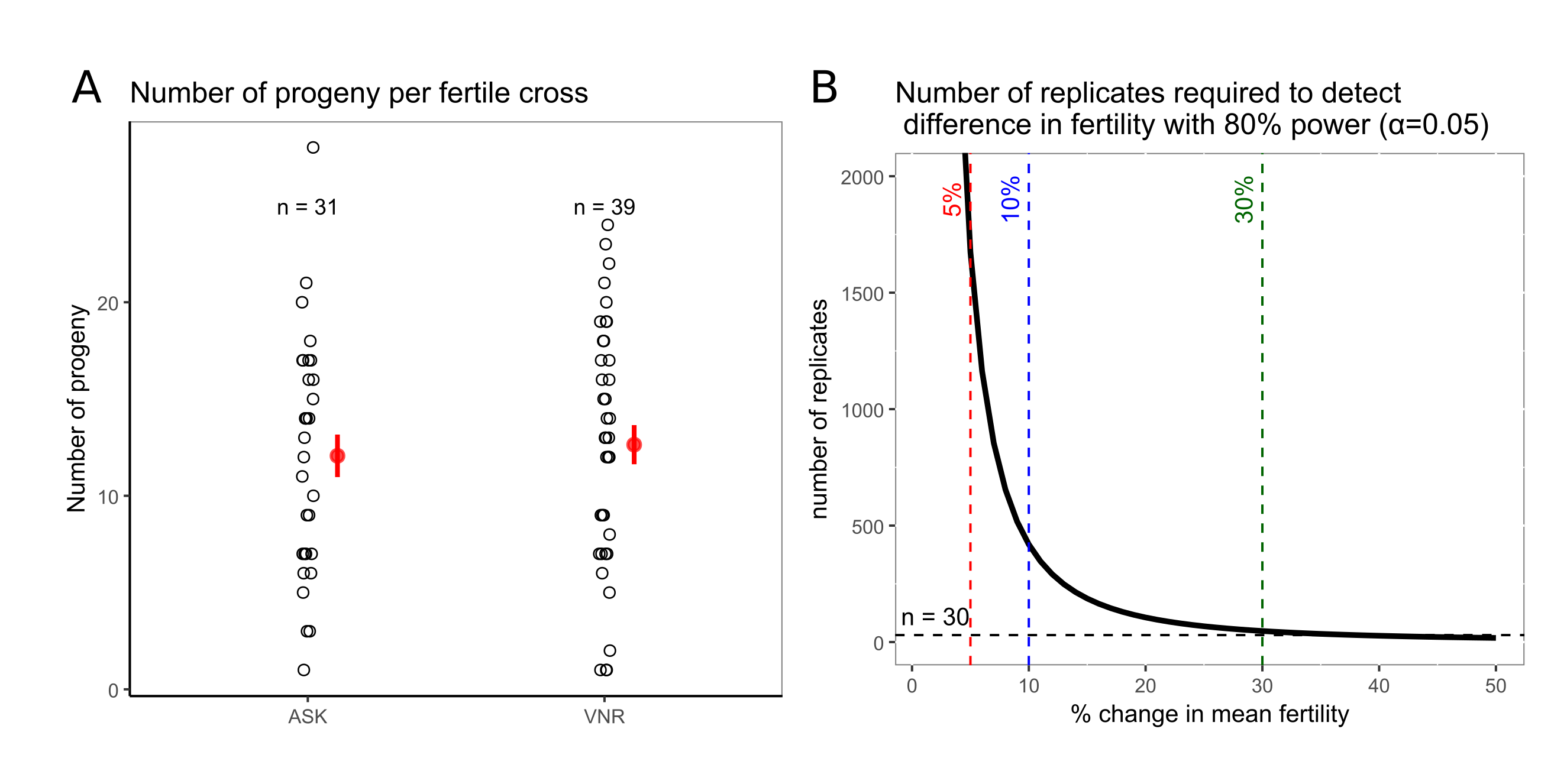

Supplement: S6 Fig — (A) Fertility of current (ASK) and ancestral (VNR) haplotypes. Single 1-3 day old virgin females were crossed with single 1-3 day old virgin males from the same line and allowed to mate for 24 hr. Adult offspring were counted 20 to 27 days later. Each open circle represents the number of adult progeny for each cross. Red circles and bars represent the mean and standard error, respectively. The two haplotypes do not show a significant difference (t-test, p=0.8961). (B) Power to detect fertility differences. The curve tracks the number of replicates required to detect a given difference in mean fertility between genotypes with 80% power (α =0.05). This calculation is based on the pooled standard deviation of progeny counts estimated from the ASK and VNR strains. The dashed red and blue and green vertical lines indicate 5%, 10% and 30% differences in mean progeny, respectively. The horizontal dashed green line indicates the current experimental sample size (n ~ 30). Detecting a 1-5% change in fertility, corresponding to large fitness differences, would require >1500 replicates. Scripts are available at https://github.com/fborne2/Trio_epistasis/. (TIFF) [file pgen.1012175.s009.tiff]

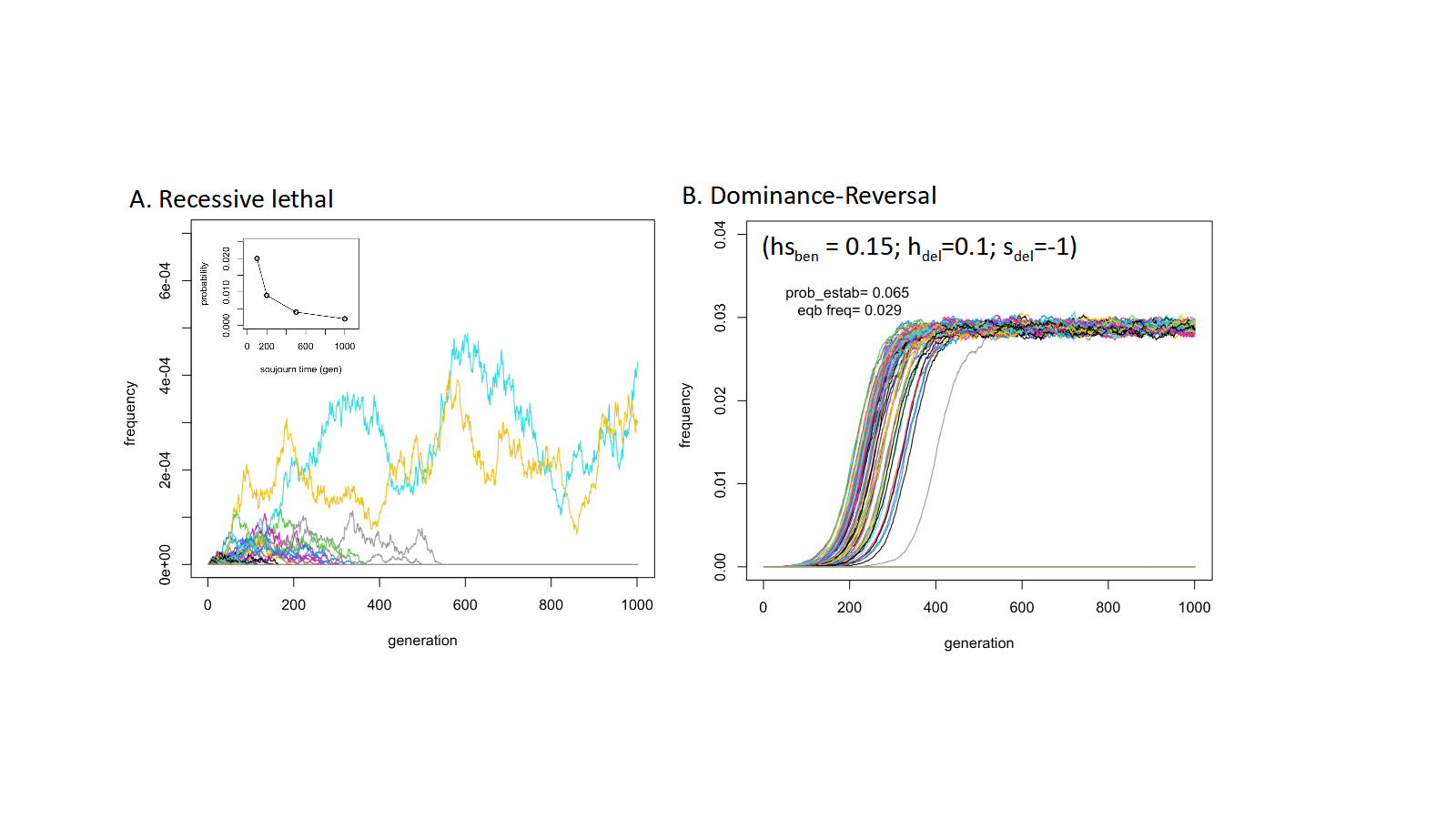

Supplement: S7 Fig — (A) Forward simulations of newly arising fully recessive (h=0) lethal mutations for a population size of 106. The inset indicates the estimated probability that a mutation will persist in the population for given sojourn time thresholds. Noteworthy is that newly arising lethals can persist in large populations for an appreciable number of generations, albeit at very low frequencies. Carter and Wagner (2022) show that in large populations like this, for modest mutation rates to secondary mutations that render the haplotype strongly beneficial, the probability of fixation can substantially exceed the neutral expectation (11). (B) Forward simulation (population size of 106) for newly arising mutations that exhibit a “dominance reversal” - in this case, a partially recessive lethal (with dominance, hdel=0.1) that has dominant beneficial effects (with hsben=0.15) on another fitness-related phenotypic axis. The resulting expected trajectory is the classic expectation for a balanced polymorphism. Prob_estab is the estimated probability of such an allele becoming established in the population, and eqb_freq is the equilibrium frequency conditional on the mutation becoming established. Modest deviations from analytical approximations are likely due to the large selection coefficients being modelled. Noteworthy is that, conditional on becoming established, such mutations can persist indefinitely at appreciable frequencies, and long enough for secondary mutations that render the haplotype neutral or beneficial. In the case of a secondary mutation that renders the haplotype neutral, the probability of fixation will be approximately equal to the equilibrium frequency and substantially higher if net beneficial. Scripts are available at https://github.com/fborne2/Trio_epistasis/. (TIFF) [file pgen.1012175.s010.tif]
