# Supplementary material for: Epistasis among clustered lineage-specific amino acid substitutions in the Drosophila Trio protein
Source: PLoS Genet. 2026 Jun 3;22(6):e1012175. doi: 10.1371/journal.pgen.1012175 (PMC13258152; doi:10.1371/journal.pgen.1012175)
Supplement: Attachment — Submitted filename: Response_to_Reviewers_Borne2026.pdf [file pgen.1012175.s012.pdf]

We thank the editor and the three reviewers for their constructive and valuable comments. We believe that we have addressed all points carefully and thoroughly, as detailed in our point-by-point response below. All changes to the manuscript are visible in tracked changes.

The revised manuscript includes substantial updates to the Results and Discussion sections, incorporating the reviewers' suggestions. In particular, we have clarified the evidence for signatures of positive selection (see also new Fig. S4), discussed the potential role of multi-nucleotide changes, and the potential epistatic effects both within the gene and across the genome. We have incorporated the recommended citations on theoretical work concerning segregating deleterious substitutions, which are now accompanied by a new supplementary figure (Fig. S7). In addition, we performed new crosses to assess the viability of intermediate F1 genotypes. These results are now included in the Results section and summarized in a new supplementary table (Table S3).

Beyond these major revisions, we have improved clarity and precision throughout the manuscript. Specifically, we revised Figures 1 and 2, modified one paragraph in the Introduction, and updated Fig. S2, Fig. S3, and Fig. S6.

Reviewer #1:

Essential protein functions require coordinated effects of amino acids, but relatively little is known about the evolutionary dynamics through which such epistatically interacting combinations arise, especially in the in vivo context of multicellular organisms. To address this knowledge gap, this paper identified a putative adaptively evolved cluster of three amino acids in D. melanogaster using a selection test and functionally characterized the effects on organismal viability of intermediates during the formation of this cluster with precisely constructed transgenic lines. They find that the ancestral (VNR) and derived (ASK) combinations of amino acids are functionally equivalent with respect to effects on viability but all intermediate combinations produce deleterious phenotypes when homozygous. From these results, the authors conclude that alleles with the individual derived amino acids initially persisted because their deleterious effects were masked in heterozygotes and then all three were able to recombine to form the derived haplotype, at which point the haplotype containing all three derived amino acid states swept to fixation.

This paper investigates a very interesting question and is well-written with clear figures. However, I have significant concerns about several of its foundations, which may invalidate the downstream inferences about the evolutionary dynamics of epistatically interacting protein residues.

1. Adaptive evolution in the system
The paper seeks to explain lineage-specific adaptive amino acid substitutions but the case for adaptive evolution in this system is at best ambiguous. The ancestral and derived amino combinations of amino acids at the three focal residues appear functionally equivalent. The authors use a site-specific selection test to identify a putative signature of selection based on clustering, but it is worth noting that the D. simulans lineage has experienced almost as many substitutions as the D. melanogaster lineage (5 vs 6 in protein). As the authors note, the

substitutions are biochemically conservative. No other types of tests of selection (selective footprints, dn/ds with more phylogenetic sampling, normal MK) are provided. I recognize that no test of selection is perfect, and the number of changes of course does not indicate anything about the functional significance of the changes because a single change can (and often does) drive adaptation. Nevertheless, alongside the absence of any molecular/biological case for why this region might be evolving adaptively in D. melanogaster (and not D. simulans), the substitution patterns present do not make a compelling case for lineage-specific adaptation in this system.

We agree that we cannot be certain that the substitutions we observe are adaptive. Even when multiple tests reject neutral models, the specific substitutions driving those signals typically remain unidentified. The only way around this criticism is to limit functional studies to the few proteins and contexts where the adaptive significance of amino acid changes are known (and we have done that in other work on Na,K-ATPase). Still, we feel that functionally exploring a broader range of examples is informative and interesting, particularly examples of adaptive protein evolution for which the only evidence for adaptation is based on statistical inference of positive selection.

Amino acid changes elsewhere in *D. simulans* Trio protein do not undermine evidence for adaptation in the *D. melanogaster* lineage. The DHPH domain is conserved in *D. simulans* and more distant species (e.g., *D. yakuba*), indicating strong constraint. Consistent with this, among 947 *D. melanogaster* sequences we observed only four nonsynonymous singletons in this domain. Even conservative substitutions are notable in such a constrained region, and the combination of rare within-population variation and fixed between-lineage differences is precisely the signal targeted by region-specific MK methods (e.g., MASS-PRF).

Further, there is strong evidence for recurrent adaptive amino acid substitutions across *Drosophila* proteins, including selective sweeps associated with these changes. Against this background, interpreting site-specific MK signals as likely reflecting adaptive protein evolution is a reasonable starting point – though we agree that alternatives should be considered.

Following the reviewer's suggestion, we performed a whole-protein MK test for Trio, which was not significant. We also found no evidence of a recent selective sweep or positive selection using branch-site models across the *Drosophila* phylogeny. However, lack of significance does not exclude adaptation, as these approaches have limited power when selection is weak, not recent, lineage-specific, or confined to few sites. Sweep-based tests, whole-protein dN/dS, and MK tests are particularly underpowered under such conditions. In contrast, site-specific MK methods (e.g., MASS-PRF) are better suited to detect adaptive protein evolution when selection is heterogeneous across proteins, motivating our use of this approach.

To acknowledge the reviewer's concern, we removed "adaptive" from the title and changed "adaptive" to "putatively adaptive" throughout the manuscript. We also added more information about the alternative tests that we performed on Trio in the results section and added a supplementary figure (Fig S4). We further added a paragraph acknowledging that our candidate clusters could be compensatory or neutral in the Discussion section (see also response to Reviewer 3 point 3).

Finally, whether the specific cluster of amino acid substitutions in *D. melanogaster* Trio are truly adaptive, merely compensatory, or the result of a multi-nucleotide mutation, does not ultimately matter for the overall impact of the paper. In our view, beginning to tackle the mechanistic details underlying MK-test signatures of positive selection, almost all of which resides in random proteins with unknown roles in fitness, is an important step toward moving the field forward.

2. Probability of evolutionary scenario.
An interesting, provocative idea put forward in the paper is that mutations that are individually deleterious and recessive segregate in the population until they recombine by chance into the same haplotype. That haplotype then confers some adaptive benefit to the organism and goes to fixation. Such a scenario playing out involving three mutations that are deleterious in isolation and in pairwise combinations seems incredibly unlikely, especially since there is no evidence that the final haplotype is adaptive. The authors could more formally explore this and simulate the likelihood of such a scenario using recombination rates, dominance, and selective coefficients calculated from their data. The authors show that intramolecular interactions are important for maintaining organismal fitness, and they demonstrate that epistasis creates incompatibilities between amino acid states present in orthologous sequences; they do not show how such epistatic clusters arise, in a molecularly or evolutionarily mechanistic framework, to increase organismal fitness.

We agree that simulations demonstrating the plausibility of such evolutionary trajectories would be informative. We note that this question has been explored extensively in prior work, and we appreciate that Reviewer 3 highlighted several relevant studies that we could include (see Reviewer 3 point 1). These studies support that this scenario –when intermediate deleterious haplotypes persist long enough for another mutation to occur that restores fitness and brings the final haplotype to fixation– could represent an important evolutionary pathway in large populations. Given that entire studies have been devoted to exploring such scenarios, a thorough and convincing treatment of this question would extend beyond the scope of our empirical study. However, we nonetheless performed a few illustrative simulations to support our claim that deleterious mutations can be maintained for many generations in large populations, particularly in the case of a dominance-reversal scenario, providing an opportunity for a second mutation or a recombination event to occur. These simulations can be found in the supplementary information (Fig S7).

We have now incorporated these references and added a paragraph to the Discussion section to more appropriately situate our results within this broader literature.

We also discussed an alternative scenario suggested by Reviewer 3, in which the three mutations could result from a single mutational event (See Reviewer 3 point 2).

3. Epistasis beyond the three residues.
The fact that all intermediates are deleterious is surprising and may represent an artifact that

impacts downstream inferences. In many of the cited papers that have found intramolecular epistasis, there are potentiating or permissive substitutions that are by themselves neutral which open up evolutionary paths for subsequent adaptive mutations. The evolutionary paths in this paper have no such steps; all intermediate steps are deleterious. While it is possible that each intermediate form was deleterious, it is more probable that epistasis from one of the other 8 fixed differences between the species is shaping the observed patterns. As an example, when any substitution from the D. simulans lineage is introduced into the D. melanogaster allele, it may produce a deleterious effect because the initial fixation of that variant in D. simulans may have required a permissive substitution that is absent when the residues are put into melanogaster. Any of the 8 variants elsewhere in the protein could affect allosteric regulation, sterics of specific conformations, or a more general feature such as stability. Recombinant alleles, in principle, could be used to show that this is not the case. In absence of such data, the presence of epistatic modifiers in the protein beyond the three sites is as or more probable than the evolutionary scenario the authors articulate. Again, it is hard to imagine three different deleterious variants arising, persisting in a population long enough to recombine into the same haplotype (when pairwise combinations are deleterious), and then the haplotype sweeping to fixation.

We agree that we cannot exclude epistasis with other parts of the protein. We added a paragraph in the discussion section noting this caveat.

In the case of Trio, it is worth emphasizing that only three additional substitutions arose in the *D. melanogaster* lineage that could potentially be relevant. These occur in regions where *D. simulans* also carry substitutions and that are less conserved across more distant species compared to other regions of the protein. This pattern makes them less likely to represent *D. melanogaster*-specific changes that interact with the focal cluster. While we cannot exclude their involvement, if such interactions do exist, they would further support—rather than weaken—our conclusion that epistasis played an important role in the fixation of the *D. melanogaster*-specific cluster.

Further, we are limited by the tractability of experiments. For this study, we have generated 7 engineered lines to evaluate all possible paths for the three focal amino acid substitutions. Reconstructing all possible trajectories that include the three additional substitutions would require generating 56 more engineered lines. This represents a substantial experimental effort, without assurance that the results would definitively substantially strengthen the study. Even then, this would not fully exclude the possibility of epistasis involving other regions of the genome.

Reviewer #2:

Comments on Borne et al. “Epistasis among clustered lineage-specific…”

In this interesting manuscript the authors investigate a cluster of 3 amino acid substitutions that have occurred along the D. melanogaster lineage. These amino acid substitutions are inferred to have been fixed by positive selection. They recreate all the intermediate states between the inferred ancestral and the Dmel state. They find that all intermediates are deleterious in homozygous form in at least one of their fitness assays. However, the intermediates are recessive, when heterozygous with the ancestral allele. This suggests a model by which the mutations might have risen to moderate frequency in the population and then recombined onto the same haplotype, which then swept to fixation; or one of the mutations rose to moderate frequency and the other mutations occurred on that background.

These are interesting results, and I don’t have any substantial comments.

1. The heterozygous effects are always tested alongside the ancestral allele; this is reasonable since under their model, the ancestral allele may well have been the dominant haplotype until the derived haplotype was formed. However, it would have been interesting to explore the fitness of a haplotype and the haplotype from which it was derived – e.g. what is the fitness of the ASR/VSR individual? This might reveal additional pathways that could have been taken.

This is a great point and we thank the reviewer for this suggestion. Notably, ANR and VSK are viable genotypes with locomotor defects that may have small effects on fitness. We thus performed ANR × ASR, ANR × ASK and VNK × VSK crosses and found that the F1 offspring are indeed viable. More generally, we expect that all heterozygotes involving ANR and VSK are likely to be viable, which could indeed open additional evolutionary pathways, whether by subsequent mutation or recombination.

We now mention these results and their implications in the Results and Discussion sections and created a supplementary Table (Table S3) with the details of these crosses.

2. Lines 207-208 “6 males and 1 female out of 113 adults” is unclear.

We agree and we modified the sentence to improve clarity:
We changed: “For VSR, the few homozygous individuals that do reach adulthood are sterile (6 males and 1 female out of 113 adults)”
To: “For VSR, some homozygous individuals reached adulthood (6 males and 1 female) but failed to produce progeny when presented with fertile mates (suggesting they may be sterile; see Methods).”

In the Methods, we have a more detailed descriptions of what we did:
“60 to 300 larvae, 100 to 200 pupae and 100 to 150 adults were counted for each engineered strain. For VSR, we obtained 6 adult homozygous males and 1 adult homozygous female. To estimate the fertility of those homozygous adult individuals, males were crossed individually with

one homozygous VNR virgin female and the female was crossed with one heterozygous VSR male. No progeny were observed after 21 days.”

3. A central question is how important are these sorts of trajectories? do most mutations fixe sequentially, or is the simultaneous fixation a common occurrence. This is a difficult question to answer.

We agree but we are not sure what the reviewer is asking for here. Our study is an attempt to begin asking this question, particularly in the context of diploid organisms. Indeed, we argue that sequential fixation – which has been highlighted in prokaryotic and other haploid systems – is unlikely in the case of Trio evolution in Drosophila. Our study represents an initial proof of concept that opens the door to more studies of this kind to get at the question of generality.

Reviewer #3:

In this manuscript, Borne et al search for evidence of epistatic interactions between clusters of substitutions in D. melanogaster. Specifically, the authors examine the possibility that amino acid changes that are near one another on primary sequence space may interact with one another in such a way that creates fitness valleys that should make a derived haplotype with multiple replacements harder to reach via a series of substitutions. The authors investigate this by first searching for clusters of amino acid replacements that appear to be have been positively selected, and then identifying a subset of these clusters that are tractable for in vivo fitness assays. For a proof of concept, the authors settle on a cluster of three amino acid replacements in the Trio protein, and use genome editing to examine fitness (as measured by viability and locomotion assays) for all 6 intermediate haplotypes. They find that all intermediate haplotypes show fitness defects when homozygous, with most being lethal. However, when heterozygous, all intermediate haplotypes appear to be roughly as fit as both the ancestral haplotype or the derived haplotype. The authors conclude that, rather than through a sequence of fixations, the derived haplotype may have been able to accumulate mutations while segregating at low frequency such that most individuals with these (recessive) mutations were heterozygous. While not being definitive, I think the authors' experimental results certainly are consistent with this explanation, and the paper is well written and fair in its framing of the evidence for their conclusions. However, I do have some comments that I would like to see addressed in a revision.

General comments:

1) The authors' model for how the derived Trio haplotype may have arisen in dmel appears to be identical to the "stochastic tunnel" model (e.g. https://pmc.ncbi.nlm.nih.gov/articles/PMC1470783/). There have been numerous theoretical studies about this model, and the authors should prominently discuss and cite that literature here. Intuitively, it makes sense that this phenomenon would be more likely when the intermediate haplotypes that form the fitness valley are recessive, as the authors find for Trio and include in their proposed version of this model. I note that while most of the papers on the stochastic tunnel phenomenon do not discuss the role of dominance (e.g. https://pubmed.ncbi.nlm.nih.gov/16050095/), some do explicitly examine the case when the deleterious effects of intermediate haplotpyes are recessive (https://pubmed.ncbi.nlm.nih.gov/12028779/).

We thank the reviewer for drawing our attention to these relevant studies. We agree that they are highly pertinent to our work, and we have now incorporated them into the discussion section and discussed our results in light of these studies.

2) The authors make the point that amino acid replacements are independent events. However, when mutations are tightly clustered, a possibility that needs to be considered is multinucleotide mutation, when a single event results in multiple simultaneous mutations. Multinucleotide mutation may not be the cause of the derived Trio haplotype, but it could have played a partial role (e.g. jumping directly to an intermediate haplotype with more than one mutation. This process

could also have been involved in some of the other 900+ clusters of mutations identified by the authors. For some of these clusters, some or all of the mutations may have occurred simultaneously, which would allow for another way to cross (at least part of) any fitness valley. The authors should discuss all of this and cite the relevant literature. Here are just a few examples of papers on this topic: https://pubmed.ncbi.nlm.nih.gov/21636278/, https://pubmed.ncbi.nlm.nih.gov/23447710/, https://pubmed.ncbi.nlm.nih.gov/25079859/, and https://pmc.ncbi.nlm.nih.gov/articles/PMC6093625/

We thank the reviewer for pointing out this alternative explanation and the literature that corresponds to it. For the specific case of Trio, we think that it is unlikely that a single mutational event would generate multiple substitutions that are each strongly deleterious on their own but jointly neutral or beneficial, though not impossible. However, we agree that this is a valid alternative explanation that should be noted and could also be involved in our other candidate clusters.

We added a paragraph discussing multi-nucleotide mutational events as a possible explanation to the establishment of clusters.

3) There are several places where the authors claim that they identified clusters of positively selected mutations. I think that, multinucleotide mutations aside, the results presented in this manuscript suggest an alternative possibility: the derived haplotype does not confer higher fitness than the ancestral haplotype, but the occurrence of compensatory substitutions creates a false signal of positive selection. It would be worth noting that this could be happening in some of their clusters, and the authors may wish to rephrase when referring to their clusters as "adaptive substitutions" (e.g. "putatively adaptive substitutions" or similar). Here is one paper I could find that is relevant to this possibility: https://academic.oup.com/mbe/article/37/11/3353/5867919

We agree with the reviewer that some of our candidate clusters could involve compensatory and non-adaptive evolution. For the specific case of Trio, it seems implausible that three individually highly deleterious mutations have a net effect that is effectively neutral but it can't be ruled out either.

Notably, while we do have the population genetic signature of positive selection, we admittedly have no direct evidence for a fitness benefit. We thus dropped “adaptive from the title and changed “adaptive” to “putatively adaptive” throughout the manuscript.

We emphasized why we think Trio might be truly adaptive in the Results section and added a supplementary figure with the results of more selection tests (see also Reviewer 1 point 1).

We added a paragraph to acknowledge that our candidate clusters could be compensatory in the Discussion section.

Specific comments:

Line 100: The phrasing here implies that pleiotropy is limited to multicellular organisms.

We removed the term pleiotropy from this sentence.

Lines 283-285: Here the authors state that they are the first to test the effects of purportedly adaptive substitutions in multicellular organisms. While their exact phrasing might make this statement technically true, I think they should cite this paper and point out that it did something fairly similar: https://www.nature.com/articles/s41559-016-0025

We corrected the sentence, including the suggested reference.

Lines 311-320: In the context of dominance reversals, the authors could cite this paper which has implications for the rate of adaptation: https://pubmed.ncbi.nlm.nih.gov/35213740/

We modified the discussion part on dominance reversals and included the reference mentioned.

HYPERLINK "https://urldefense.com/v3/__https://pubmed.ncbi.nlm.nih.gov/35213740/__;!!BDUfV1Et5lrpZQ!Xrs1iPg0JAi8DXVl9hgx1hvNZOlFsiQlMA1fDBg-S8oiJLLuMbkgoiSk8-mD9O2e7qHEZmdp4G0eDS4ysiyp8Yk$"Lines 338-341: Here the authors are discussing how the simultaneous fixation of multiple alleles might cause one to incorrectly conclude that the reduction in diversity caused by these fixations was cumulative from multiple independent substitutions rather than the fixation of a single haplotype. I think I know the type of study the authors are referring to here but the description is somewhat vague, so it would be helpful to cite some example studies.

This is indeed the point we aim to highlight. Our reasoning is primarily intuitive and stems from classical models of selective sweeps and genetic hitchhiking, which assume independence among sites and sequential fixation. To our knowledge, the consequences of simultaneous fixation of multiple mutations for inferred diversity reductions have not been explicitly investigated. We have clarified this point in the revised manuscript by explicitly referring to classical hitchhiking models and by adding a citation to Fay et al. (2011), which discusses related issues of epistasis in the context of the McDonald–Kreitman test.

Lines 394-399: To clarify, the authors are saying that there were 940 clusters of at least 2 substitutions within a 20-codon window (call this set A), and then some subset of these had only 2 or 3 substitutions within the 20-codon window (call this set B). The clusters in set B were found in 679 genes. Is this interpretation correct? If so, can the authors share here 1) how many genes are account for the clusters in set A, and 2) how many clusters are in set B?

We agree that the methods were not entirely clear. This interpretation is not completely correct:

Set A: 940 protein fragments (corresponding to 919 proteins) contain at least 1 cluster (defined by at least 2 substitutions within a 20-codon window). This set A contains a total of 1116 clusters.

Set B: 691 protein fragments (corresponding to 679 proteins) contain at least 1 experimentally tractable cluster (defined by 2 or 3 substitutions within 20-codon windows). This set B contains a total of 795 clusters.

The list of the proteins and the coordinates of the clusters can be found here: https://github.com/fborne2/Trio_epistasis/tree/main/MASS-PRF_analysis/results

We corrected the Methods section:

"Of the 5355 proteins for which runs were successful, 1144 had at least one site under positive selection. Among them, 919 proteins harbor clustered substitutions, defined by at least two sites less than 20 codons apart. Within this set, we found that 679 proteins contain at least one potential experimentally tractable cluster, corresponding to a total of 795 clusters."
