# Supplementary material for: Epistasis among clustered lineage-specific amino acid substitutions in the Drosophila Trio protein
Source: PLoS Genet. 2026 Jun 3;22(6):e1012175. doi: 10.1371/journal.pgen.1012175 (PMC13258152; doi:10.1371/journal.pgen.1012175)
Supplement: Peer review history

# Peer review: Decision Letter 0

**DOI:** 10.1371/journal.pgen.1012175.r001

**Authors:** Kelly Dyer

30 Jan 2026

PGENETICS-D-25-01120

Epistasis among clustered lineage-specific adaptive amino acid substitutions in the Drosophila Trio protein.

PLOS Genetics

Dear Dr. Borne,

Thank you for submitting your manuscript to PLOS Genetics. After careful consideration, we feel that it has merit but does not fully meet PLOS Genetics's publication criteria as it currently stands. Therefore, we invite you to submit a revised version of the manuscript that addresses the points raised during the review process.

Please submit your revised manuscript by Mar 31 2026 11:59PM. If you will need more time than this to complete your revisions, please reply to this message or contact the journal office at plosgenetics@plos.org. When you're ready to submit your revision, log on to https://www.editorialmanager.com/pgenetics/ and select the 'Submissions Needing Revision' folder to locate your manuscript file.

Please include the following items when submitting your revised manuscript:

* A letter that responds to each point raised by the editor and reviewer(s). You should upload this letter as a separate file labeled 'Response to Reviewers'. This file does not need to include responses to any formatting updates and technical items listed in the 'Journal Requirements' section below.

* A marked-up copy of your manuscript that highlights changes made to the original version. You should upload this as a separate file labeled 'Revised Manuscript with Track Changes'.

* An unmarked version of your revised paper without tracked changes. You should upload this as a separate file labeled 'Manuscript'.

If you would like to make changes to your financial disclosure, competing interests statement, or data availability statement, please make these updates within the submission form at the time of resubmission. Guidelines for resubmitting your figure files are available below the reviewer comments at the end of this letter.

We look forward to receiving your revised manuscript.

Kind regards,

Colin Meiklejohn

Academic Editor

PLOS Genetics

Kelly Dyer

Section Editor

PLOS Genetics

Aimée Dudley

Editor-in-Chief

PLOS Genetics

Anne Goriely

Editor-in-Chief

PLOS Genetics

**Additional Editor Comments:**

Thank you very much for your patience with this lengthy review process. Your manuscript has been evaluated by three reviewers, all of whom appreciated the value of your study. Please address the reviewers' comments, in particular consider incorporating the suggested citations from reviewer 2, the potential role of multi-nucleotide mutations in the evolution of the derived haplotype, and the possibility that intramolecular epistasis at divergent sites excluding the three positions investigated here may modulate the deleterious effects of intermediate genotypes.

**Journal Requirements:**

1) Please ensure that the CRediT author contributions listed for every co-author are completed accurately and in full.

At this stage, the following Authors/Authors require contributions: Flora Borne, Andrew M. Taverner, and Peter Andolfatto. Please ensure that the full contributions of each author are acknowledged in the "Add/Edit/Remove Authors" section of our submission form.

The list of CRediT author contributions may be found here: https://journals.plos.org/plosgenetics/s/authorship#loc-author-contributions

2) We ask that a manuscript source file is provided at Revision. Please upload your manuscript file as a .doc, .docx, .rtf or .tex. If you are providing a .tex file, please upload it under the item type u2018LaTeX Source Fileu2019 and leave your .pdf version as the item type u2018Manuscriptu2019.

3) We do not publish any copyright or trademark symbols that usually accompany proprietary names, eg ©,  ®, or TM  (e.g. next to drug or reagent names). Therefore please remove all instances of trademark/copyright symbols throughout the text, including:

- ® on page: 23.

4) Please upload all main figures as separate Figure files in .tif or .eps format. For more information about how to convert and format your figure files please see our guidelines:

https://journals.plos.org/plosgenetics/s/figures

5) We have noticed that you have uploaded Supporting Information files, but you have not included a list of legends. Please add a full list of legends for your Supporting Information files after the references list.

6) Please amend your detailed Financial Disclosure statement. This is published with the article. It must therefore be completed in full sentences and contain the exact wording you wish to be published.

- State the initials, alongside each funding source, of each author to receive each grant. For example: "This work was supported by the National Institutes of Health (####### to AM; ###### to CJ) and the National Science Foundation (###### to AM)."

- State what role the funders took in the study. If the funders had no role in your study, please state: "The funders had no role in study design, data collection and analysis, decision to publish, or preparation of the manuscript.".

If you did not receive any funding for this study, please simply state: u201cThe authors received no specific funding for this work.u201d

**Reviewers' comments:**

Reviewer's Responses to Questions

**Comments to the Authors:**

**Please note here if the review is uploaded as an attachment.**

Reviewer #1: Essential protein functions require coordinated effects of amino acids, but relatively little is known about the evolutionary dynamics through which such epistatically interacting combinations arise, especially in the in vivo context of multicellular organisms. To address this knowledge gap, this paper identified a putative adaptively evolved cluster of three amino acids in D. melanogaster using a selection test and functionally characterized the effects on organismal viability of intermediates during the formation of this cluster with precisely constructed transgenic lines. They find that the ancestral (VNR) and derived (ASK) combinations of amino acids are functionally equivalent with respect to effects on viability but all intermediate combinations produce deleterious phenotypes when homozygous. From these results, the authors conclude that alleles with the individual derived amino acids initially persisted because their deleterious effects were masked in heterozygotes and then all three were able to recombine to form the derived haplotype, at which point the haplotype containing all three derived amino acid states swept to fixation.

This paper investigates a very interesting question and is well-written with clear figures. However, I have significant concerns about several of its foundations, which may invalidate the downstream inferences about the evolutionary dynamics of epistatically interacting protein residues.

1. Adaptive evolution in the system

The paper seeks to explain lineage-specific adaptive amino acid substitutions but the case for adaptive evolution in this system is at best ambiguous. The ancestral and derived amino combinations of amino acids at the three focal residues appear functionally equivalent. The authors use a site-specific selection test to identify a putative signature of selection based on clustering, but it is worth noting that the D. simulans lineage has experienced almost as many substitutions as the D. melanogaster lineage (5 vs 6 in protein). As the authors note, the substitutions are biochemically conservative. No other types of tests of selection (selective footprints, dn/ds with more phylogenetic sampling, normal MK) are provided. I recognize that no test of selection is perfect, and the number of changes of course does not indicate anything about the functional significance of the changes because a single change can (and often does) drive adaptation. Nevertheless, alongside the absence of any molecular/biological case for why this region might be evolving adaptively in D. melanogaster (and not D. simulans), the substitution patterns present do not make a compelling case for lineage-specific adaptation in this system.

2. Probability of evolutionary scenario.

An interesting, provocative idea put forward in the paper is that mutations that are individually deleterious and recessive segregate in the population until they recombine by chance into the same haplotype. That haplotype then confers some adaptive benefit to the organism and goes to fixation. Such a scenario playing out involving three mutations that are deleterious in isolation and in pairwise combinations seems incredibly unlikely, especially since there is no evidence that the final haplotype is adaptive. The authors could more formally explore this and simulate the likelihood of such a scenario using recombination rates, dominance, and selective coefficients calculated from their data. The authors show that intramolecular interactions are important for maintaining organismal fitness, and they demonstrate that epistasis creates incompatibilities between amino acid states present in orthologous sequences; they do not show how such epistatic clusters arise, in a molecularly or evolutionarily mechanistic framework, to increase organismal fitness.

3. Epistasis beyond the three residues.

The fact that all intermediates are deleterious is surprising and may represent an artifact that impacts downstream inferences. In many of the cited papers that have found intramolecular epistasis, there are potentiating or permissive substitutions that are by themselves neutral which open up evolutionary paths for subsequent adaptive mutations. The evolutionary paths in this paper have no such steps; all intermediate steps are deleterious. While it is possible that each intermediate form was deleterious, it is more probable that epistasis from one of the other 8 fixed differences between the species is shaping the observed patterns. As an example, when any substitution from the D. simulans lineage is introduced into the D. melanogaster allele, it may produce a deleterious effect because the initial fixation of that variant in D. simulans may have required a permissive substitution that is absent when the residues are put into melanogaster. Any of the 8 variants elsewhere in the protein could affect allosteric regulation, sterics of specific conformations, or a more general feature such as stability. Recombinant alleles, in principle, could be used to show that this is not the case. In absence of such data, the presence of epistatic modifiers in the protein beyond the three sites is as or more probable than the evolutionary scenario the authors articulate. Again, it is hard to imagine three different deleterious variants arising, persisting in a population long enough to recombine into the same haplotype (when pairwise combinations are deleterious), and then the haplotype sweeping to fixation.

Reviewer #2: Comments on Borne et al. “Epistasis among clustered lineage-specific…”

In this interesting manuscript the authors investigate a cluster of 3 amino acid substitutions that have occurred along the D. melanogaster lineage. These amino acid substitutions are inferred to have been fixed by positive selection. They recreate all the intermediate states between the inferred ancestral and the Dmel state. They find that all intermediates are deleterious in homozygous form in at least one of their fitness assays. However, the intermediates are recessive, when heterozygous with the ancestral allele. This suggests a model by which the mutations might have risen to moderate frequency in the population and then recombined onto the same haplotype, which then swept to fixation; or one of the mutations rose to moderate frequency and the other mutations occurred on that background.

These are interesting results, and I don’t have any substantial comments.

1. The heterozygous effects are always tested alongside the ancestral allele; this is reasonable since under their model, the ancestral allele may well have been the dominant haplotype until the derived haplotype was formed. However, it would have been interesting to explore the fitness of a haplotype and the haplotype from which it was derived – e.g. what is the fitness of the ASR/VSR individual? This might reveal additional pathways that could have been taken.

2. Lines 207-208 “6 males and 1 female out of 113 adults” is unclear.

3. A central question is how important are these sorts of trajectories? do most mutations fixe sequentially, or is the simultaneous fixation a common occurrence. This is a difficult question to answer.

Reviewer #3: In this manuscript, Borne et al search for evidence of epistatic interactions between clusters of substitutions in D. melanogaster. Specifically, the authors examine the possibility that amino acid changes that are near one another on primary sequence space may interact with one another in such a way that creates fitness valleys that should make a derived haplotype with multiple replacements harder to reach via a series of substitutions. The authors investigate this by first searching for clusters of amino acid replacements that appear to be have been positively selected, and then identifying a subset of these clusters that are tractable for in vivo fitness assays. For a proof of concept, the authors settle on a cluster of three amino acid replacements in the Trio protein, and use genome editing to examine fitness (as measured by viability and locomotion assays) for all 6 intermediate haplotypes. They find that all intermediate haplotypes show fitness defects when homozygous, with most being lethal. However, when heterozygous, all intermediate haplotypes appear to be roughly as fit as both the ancestral haplotype or the derived haplotype. The authors conclude that, rather than through a sequence of fixations, the derived haplotype may have been able to accumulate mutations while segregating at low frequency such that most individuals with these (recessive) mutations were heterozygous. While not being definitive, I think the authors' experimental results certainly are consistent with this explanation, and the paper is well written and fair in its framing of the evidence for their conclusions. However, I do have some comments that I would like to see addressed in a revision.

General comments:

1) The authors' model for how the derived Trio haplotype may have arisen in dmel appears to be identical to the "stochastic tunnel" model (e.g. https://pmc.ncbi.nlm.nih.gov/articles/PMC1470783/). There have been numerous theoretical studies about this model, and the authors should prominently discuss and cite that literature here. Intuitively, it makes sense that this phenomenon would be more likely when the intermediate haplotypes that form the fitness valley are recessive, as the authors find for Trio and include in their proposed version of this model. I note that while most of the papers on the stochastic tunnel phenomenon do not discuss the role of dominance (e.g. https://pubmed.ncbi.nlm.nih.gov/16050095/), some do explicitly examine the case when the deleterious effects of intermediate haplotpyes are recessive (https://pubmed.ncbi.nlm.nih.gov/12028779/).

2) The authors make the point that amino acid replacements are independent events. However, when mutations are tightly clustered, a possibility that needs to be considered is multinucleotide mutation, when a single event results in multiple simultaneous mutations. Multinucleotide mutation may not be the cause of the derived Trio haplotype, but it could have played a partial role (e.g. jumping directly to an intermediate haplotype with more than one mutation. This process could also have been involved in some of the other 900+ clusters of mutations identified by the authors. For some of these clusters, some or all of the mutations may have occurred simultaneously, which would allow for another way to cross (at least part of) any fitness valley. The authors should discuss all of this and cite the relevant literature. Here are just a few examples of papers on this topic: https://pubmed.ncbi.nlm.nih.gov/21636278/, https://pubmed.ncbi.nlm.nih.gov/23447710/, https://pubmed.ncbi.nlm.nih.gov/25079859/, and https://pmc.ncbi.nlm.nih.gov/articles/PMC6093625/

3) There are several places where the authors claim that they identified clusters of positively selected mutations. I think that, multinucleotide mutations aside, the results presented in this manuscript suggest an alternative possibility: the derived haplotype does not confer higher fitness than the ancestral haplotype, but the occurrence of compensatory substitutions creates a false signal of positive selection. It would be worth noting that this could be happening in some of their clusters, and the authors may wish to rephrase when referring to their clusters as "adaptive substitutions" (e.g. "putatively adaptive substitutions" or similar). Here is one paper I could find that is relevant to this possibility: https://academic.oup.com/mbe/article/37/11/3353/5867919

Specific comments:

Line 100: The phrasing here implies that pleiotropy is limited to multicellular organisms.

Lines 283-285: Here the authors state that they are the first to test the effects of purportedly adaptive substitutions in multicellular organism. While their exact phrasing might make this statement technically true, I think they should cite this paper and point out that it did something fairly similar: https://www.nature.com/articles/s41559-016-0025

Lines 311-320: In the context of dominance reversals, the authors could cite this paper which has implications for the rate of adaptation: https://pubmed.ncbi.nlm.nih.gov/35213740/

Lines 338-341: Here the authors are discussing how the simultaneous fixation of multiple alleles might cause one to incorrectly conclude that the reduction in diversity caused by these fixations was cumulative from multiple independent substitutions rather than the fixation of a single haplotype. I think I know the type of study the authors are referring to here but the description is somewhat vague, so it would be helpful to cite some example studies.

Lines 394-399: To clarify, the authors are saying that there were 940 clusters of at least 2 substitutions within a 20-codon window (call this set A), and then some subset of these had only 2 or 3 substitutions within the 20-codon window (call this set B). The clusters in set B were found in 679 genes. Is this interpretation correct? If so, can the authors share here 1) how many genes are account for the clusters in set A, and 2) how many clusters are in set B?

**********

**Have all data underlying the figures and results presented in the manuscript been provided?**

Large-scale datasets should be made available via a public repository as described in the *PLOS Genetics* data availability policy, and numerical data that underlies graphs or summary statistics should be provided in spreadsheet form as supporting information.

Reviewer #1: Yes

Reviewer #2: Yes

Reviewer #3: None

**********

PLOS authors have the option to publish the peer review history of their article (what does this mean?). If published, this will include your full peer review and any attached files.

If you choose “no”, your identity will remain anonymous but your review may still be made public.

**Do you want your identity to be public for this peer review?** For information about this choice, including consent withdrawal, please see our Privacy Policy.

Reviewer #1: No

Reviewer #2: No

Reviewer #3: No

[NOTE: If reviewer comments were submitted as an attachment file, they will be attached to this email and accessible via the submission site. Please log into your account, locate the manuscript record, and check for the action link "View Attachments". If this link does not appear, there are no attachment files.]

**Figure resubmission:**

-->While revising your submission, we strongly recommend that you use PLOS’s NAAS tool (https://ngplosjournals.pagemajik.ai/artanalysis) to test your figure files. NAAS can convert your figure files to the TIFF file type and meet basic requirements (such as print size, resolution), or provide you with a report on issues that do not meet our requirements and that NAAS cannot fix.

After uploading your figures to PLOS’s NAAS tool - https://ngplosjournals.pagemajik.ai/artanalysis, NAAS will process the files provided and display the results in the "Uploaded Files" section of the page as the processing is complete. If the uploaded figures meet our requirements (or NAAS is able to fix the files to meet our requirements), the figure will be marked as "fixed" above. If NAAS is unable to fix the files, a red "failed" label will appear above. When NAAS has confirmed that the figure files meet our requirements, please download the file via the download option, and include these NAAS processed figure files when submitting your revised manuscript.

**Reproducibility:**

To enhance the reproducibility of your results, we recommend that authors of applicable studies deposit laboratory protocols in protocols.io, where a protocol can be assigned its own identifier (DOI) such that it can be cited independently in the future. Additionally, PLOS ONE offers an option to publish peer-reviewed clinical study protocols. Read more information on sharing protocols at https://plos.org/protocols?utm_medium=editorial-email&utm_source=authorletters&utm_campaign=protocols

# Author comment: Author response to Decision Letter 1

**DOI:** 10.1371/journal.pgen.1012175.r002

7 Apr 2026

Attachment Submitted filename: Response_to_Reviewers_Borne2026.pdf

# Peer review: Decision Letter 1

**DOI:** 10.1371/journal.pgen.1012175.r003

**Authors:** Kelly Dyer

21 May 2026

Dear Dr Borne,

We are pleased to inform you that your manuscript entitled "Epistasis among clustered lineage-specific amino acid substitutions in the Drosophila Trio protein." has been editorially accepted for publication in PLOS Genetics. Congratulations!  We encourage you to consider the final comment from Reviewer #1 about the word "adaptive" in the legend of Figure 3 and suggest "evolutionary" or "non-deleterious" as alternative descriptions of these paths.

Before your submission can be formally accepted and sent to production you will need to complete our formatting changes, which you will receive in a follow up email. Please be aware that it may take several days for you to receive this email; during this time no action is required by you. Please note: the accept date on your published article will reflect the date of this provisional acceptance, but your manuscript will not be scheduled for publication until the required changes have been made.

Once your paper is formally accepted, an uncorrected proof of your manuscript will be published online ahead of the final version, unless you’ve already opted out via the online submission form. If, for any reason, you do not want an earlier version of your manuscript published online or are unsure if you have already indicated as such, please let the journal staff know immediately at plosgenetics@plos.org.

In the meantime, please log into Editorial Manager at https://www.editorialmanager.com/pgenetics/, click the "Update My Information" link at the top of the page, and update your user information to ensure an efficient production and billing process. Note that PLOS requires an ORCID iD for all corresponding authors. Therefore, please ensure that you have an ORCID iD and that it is validated in Editorial Manager. To do this, go to ‘Update my Information’ (in the upper left-hand corner of the main menu), and click on the Fetch/Validate link next to the ORCID field.  This will take you to the ORCID site and allow you to create a new iD or authenticate a pre-existing iD in Editorial Manager.

If you have a press-related query, or would like to know about making your underlying data available (as you will be aware, this is required for publication), please see the end of this email. If your institution or institutions have a press office, please notify them about your upcoming article at this point, to enable them to help maximise its impact. Inform journal staff as soon as possible if you are preparing a press release for your article and need a publication date.

Thank you again for supporting open-access publishing; we are looking forward to publishing your work in PLOS Genetics!

Yours sincerely,

Colin Meiklejohn

Academic Editor

PLOS Genetics

Kelly Dyer

Section Editor

PLOS Genetics

Aimée Dudley

Editor-in-Chief

PLOS Genetics

Anne Goriely

Editor-in-Chief

PLOS Genetics

www.plosgenetics.org

BlueSky: @plos.bsky.social

----------------------------------------------------

Comments from the reviewers (if applicable):

Reviewer's Responses to Questions

**Comments to the Authors:**

**Please note here if the review is uploaded as an attachment.**

Reviewer #1: The authors have made numerous substantive changes throughout the manuscript that have enriched it and have addressed the issues previously raised. The added data for heterozygous genotypes, in particular, strengthen inferences about the plausible evolutionary paths from the ancestral VNR to the derived ASK allele.

My only remaining critique concerns Figure 3. I think it is difficult to make the case that Figure 3 shows an adaptive path; fitness remains flat for the heterozygous genotypes with intermediate alleles. I would simply relabel this as “evolutionary paths.” There may indeed be conditions under which the seemingly neutral or deleterious variants were adaptive, and it is reasonable to speculate about this. However, the data show that no allele increases fitness relative to VNR; as such, they seem more consistent with a neutral or compensatory “rewiring” of functional residues, akin to observations from regulatory circuit evolution (e.g., Johnson et al. 2017, https://doi.org/10.1016/j.gde.2017.09.004). The evolutionary path(s) the authors uncover are interesting regardless of whether the amino acid changes were adaptive. The findings --and testing of molecular hypotheses at the organismal level -- raise important issues for how insights from protein epistasis studies—largely drawn from haploid or in vitro contexts—should inform our understanding of the population genetic processes through which alleles segregate and fix.

Reviewer #2: The authors have satisfactorily addressed my questions. This is an interesting manuscript which I look forward to seeing in print.

Reviewer #3: The authors have thoroughly addressed my comments.

**********

**Have all data underlying the figures and results presented in the manuscript been provided?**

Large-scale datasets should be made available via a public repository as described in the *PLOS Genetics* data availability policy, and numerical data that underlies graphs or summary statistics should be provided in spreadsheet form as supporting information.

Reviewer #1: None

Reviewer #2: Yes

Reviewer #3: Yes

**********

PLOS authors have the option to publish the peer review history of their article (what does this mean?). If published, this will include your full peer review and any attached files.

If you choose “no”, your identity will remain anonymous but your review may still be made public.

**Do you want your identity to be public for this peer review?** For information about this choice, including consent withdrawal, please see our Privacy Policy.

Reviewer #1: No

Reviewer #2: No

Reviewer #3: No

----------------------------------------------------

**Data Deposition**

If you have submitted a Research Article or Front Matter that has associated data that are not suitable for deposition in a subject-specific public repository (such as GenBank or ArrayExpress), one way to make that data available is to deposit it in the Dryad Digital Repository. As you may recall, we ask all authors to agree to make data available; this is one way to achieve that. A full list of recommended repositories can be found on our website.

The following link will take you to the Dryad record for your article, so you won't have to re‐enter its bibliographic information, and can upload your files directly:

http://datadryad.org/submit?journalID=pgenetics&manu=PGENETICS-D-25-01120R1

More information about depositing data in Dryad is available at http://www.datadryad.org/depositing. If you experience any difficulties in submitting your data, please contact help@datadryad.org for support.

Additionally, please be aware that our data availability policy requires that all numerical data underlying display items are included with the submission, and you will need to provide this before we can formally accept your manuscript, if not already present.

----------------------------------------------------

**Press Queries**

If you or your institution will be preparing press materials for this manuscript, or if you need to know your paper's publication date for media purposes, please inform the journal staff as soon as possible so that your submission can be scheduled accordingly. Your manuscript will remain under a strict press embargo until the publication date and time. This means an early version of your manuscript will not be published ahead of your final version. PLOS Genetics may also choose to issue a press release for your article. If there's anything the journal should know or you'd like more information, please get in touch via plosgenetics@plos.org.

# Editor report: Acceptance letter

**DOI:** 10.1371/journal.pgen.1012175.r004

**Authors:** Kelly Dyer

PGENETICS-D-25-01120R1

Epistasis among clustered lineage-specific amino acid substitutions in the Drosophila Trio protein.

Dear Dr Borne,

We are pleased to inform you that your manuscript entitled "Epistasis among clustered lineage-specific amino acid substitutions in the Drosophila Trio protein." has been formally accepted for publication in PLOS Genetics! Your manuscript is now with our production department and you will be notified of the publication date in due course.

The corresponding author will soon be receiving a typeset proof for review, to ensure errors have not been introduced during production. Please review the PDF proof of your manuscript carefully, as this is the last chance to correct any errors. Please note that major changes, or those which affect the scientific understanding of the work, will likely cause delays to the publication date of your manuscript.

Soon after your final files are uploaded, unless you have opted out or your manuscript is a front-matter piece, the early version of your manuscript will be published online. The date of the early version will be your article's publication date. The final article will be published to the same URL, and all versions of the paper will be accessible to readers.

For Research Articles, you will receive an invoice from PLOS for your publication fee after your manuscript has reached the completed accept phase. If you receive an email requesting payment before acceptance or for any other service, this may be a phishing scheme. Learn how to identify phishing emails and protect your accounts at https://explore.plos.org/phishing.

Thank you again for supporting PLOS Genetics and open-access publishing. We are looking forward to publishing your work!

With kind regards,

Anita Estes

PLOS Genetics

On behalf of:

The PLOS Genetics Team

Carlyle House, Carlyle Road, Cambridge CB4 3DN | United Kingdom

plosgenetics@plos.org | +44 (0) 1223-442823

plosgenetics.org | Twitter: @PLOSGenetics
